# Randomized Clinical Trial on Direct Composite and Indirect Ceramic Laminate Veneers in Multiple Diastema Closure Cases: Two-Year Follow-Up

**DOI:** 10.3390/ma17143514

**Published:** 2024-07-16

**Authors:** Ali A. Elkaffas, Abdullah Alshehri, Ali R. Alqahtani, Mohammed A. Abuelqomsan, Yahya A. M. Deeban, Refal S. Albaijan, Khalid K. Alanazi, Abdulellah F. Almudahi

**Affiliations:** 1 Department of Conservative Dental Sciences, College of Dentistry, Prince Sattam Bin Abdulaziz University, Alkharj 16278, Saudi Arabiakk.alanazi@psau.edu.sa (K.K.A.);; 2 Department of Operative Dentistry, Faculty of Dentistry, Mansoura University, Mansoura 35516, Egypt; 3 Department of Restorative and Prosthetic Dental Sciences, College of Clinical Dentistry, Majmaah University, Al-Majmaah 15341, Saudi Arabia; 4 Department of Prosthodontics, College of Dentistry, Prince Sattam Bin Abdulaziz University, Alkharj 16278, Saudi Arabia

**Keywords:** randomized clinical trial, glass ceramics, direct composite, veneers, diastema closure

## Abstract

In recent years, laminate veneer restorations should be considered as a minimally invasive treatment option for several aesthetic reasons. This study compared direct composite veneers’ and indirect ceramic laminate veneers’ longevity in multiple diastema closures. A total of 28 patients with a mean age of 26 years received 60 direct resin composite (Estelite Asteria; n = 14) and 60 indirect ceramic veneers (IPS e.max Press; n = 14) on the maxillary anterior teeth with diastema closure. Veneers were evaluated at baseline and thereafter every 6 months for up to 2 years using USPHS criteria. Data were analyzed with Fisher’s exact and chi-squared tests, while Kaplan–Meier curve was used to assess time to event. In total, three failures were observed in the form of debonding (n = 1) and fracture (n = 2) in the indirect ceramic veneers. No significant difference was observed between the survival rates of composite and ceramic veneers (Estelite Asteria: 93.4%, IPS e.max Press: 95%; *p* > 0.05). The overall survival rate was 94.2% (Kaplan–Meier). Staining (n = 11) and roughness (n = 14) were frequently observed for the resin composite veneers up to the final recall. Thereby, the preliminary results from this clinical trial comparing two veneer materials indicated that their survival rates were statistically similar. However, surface quality changes were more frequent in the composite veneer material.

## 1. Introduction

As a minimally invasive treatment alternative, laminate veneer restorations ought to be considered for several esthetic reasons. The literature suggests that there is disagreement among researchers about whether ceramic or composite materials should be employed for restorative treatment [1,2]. Some attempts have been made to compare indirect composites and indirect ceramics (IC) in vivo, but none have been made to compare direct composites (DC) and IC in diastema closure cases in vivo [3].

Ceramics are usually the material of choice because of their favorable characteristics, including better color stability and fracture resistance compared to resin composites [4,5]. Clinical investigations have shown that IC had over 90% survival rates after 4 to 10 years of follow-up [4,6,7,8,9,10]. However, clinical research on indirect resin composite laminate veneers revealed they were prone to fracture and surface discoloration [1,11].

Moreover, Thais et al.’s 10-year study found that IC outlasted composite veneers in terms of both success and survivability [12]. Meanwhile, Bora et al. found that the survival rate of direct diastema closure and recontouring of DC veneers in a 4-year clinical trial was 90% [13].

When direct restorations are made to change the tooth’s shape or position, they are referred to as DC veneers [14]. Recontouring restorations without cavity preparation and diastema closure restorations are two of the most exceptional forms of DC restorations [14]. These techniques’ key benefits include shorter treatment times, tooth structure preservation, and comparatively lower costs. Nonetheless, the most crucial factors to take into account are marginal adaptability, color matching that corresponds with the natural tooth color, and the resin composite restoration’s long-lasting nature [15]. Nevertheless, diastema closure and recontouring are operative procedures that require specific skills and are therefore operator-dependent. It is indeed difficult to achieve correct symmetry freehand in direct restorations and not every clinician can do it [16].

Additionally, the resin composite’s clinical performance concerning maxillary anterior restorations has not been well studied [14,17,18]. According to a meta-analysis, 3- to 5-year clinical trial longevity varied from 79% to 89% [19,20,21], whereas 10-year survival rates of 95% for class III and 90% for class IV were documented [22].

The purpose of this study was to gather further information in a specific research area. Hence, the current study aimed to evaluate DC laminate veneers’ longevity compared to IC laminate veneers’ longevity in multiple diastema closure cases using USPHS criteria [23]. The formulated null hypothesis was that there is no significant difference in DC and IC laminate veneers’ clinical performance in multiple diastema closure cases over 2 years.

## 2. Materials and Methods

### 2.1. Restorative Materials and Curing Device

In the current study, the manufacturer’s instructions were followed for the use of the nanofilled composite resin (Estelite Asteria, Tokuyama Dental, Tokyo, Japan) for DC and IPS e.max Press (Ivoclar Vivadent, Amherst, NY, USA) for IC. A light curing device with an output density of 655 mW/cm^2^ (LED Bluephase C5, Ivoclar Vivadent, Amherst, NY, USA) was used. Demetron LED light meters were used to measure the light curing unit’s intensity regularly (Demetron Research Corp., Danbury, CT, USA). Table 1 shows the brand name, description, chemical composition, and manufacturers of the materials.

### 2.2. Study Design, Blinding, and Randomization

The Consolidated Standards of Reporting Trials statement was adhered to in the description of the experimental design [24]. This study was a randomized controlled clinical trial that was double-blinded for both trial participants and outcome assessors. Participants were blind to design, hypotheses, eligibility criteria, and outcomes. However, outcome assessors were blind to hypotheses, group assignment, purpose, and interventions along the follow-up period. A coin toss was used to determine the choice of material in the randomization process. Through computerized sequence generation (www.randomizer.org) accessed on 10 October 2021, participants were separated into 2 groups using a 1:1 allocation ratio.

### 2.3. Patient Selection and Eligibility Criteria

**Figure 1:**
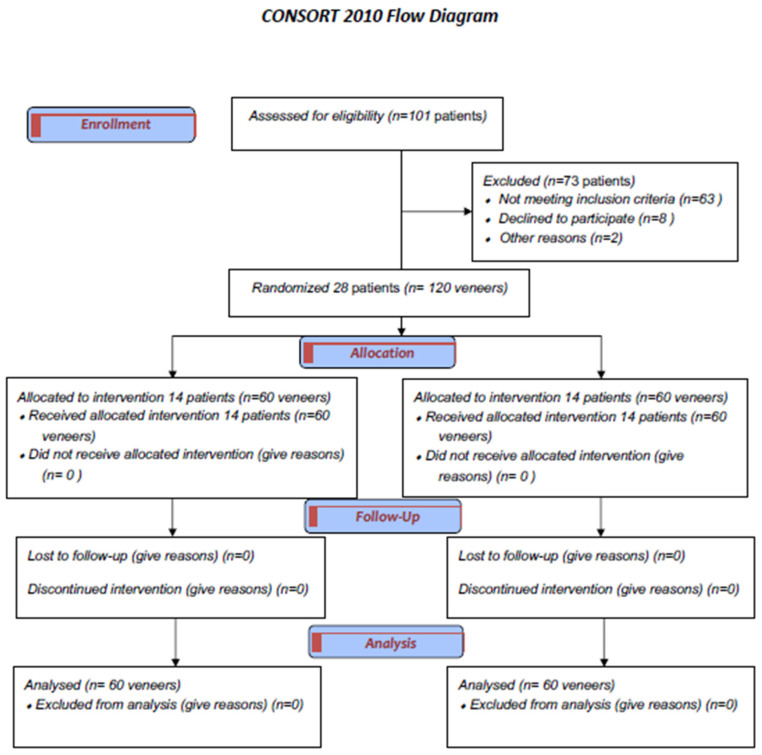
Consort flow chart presenting the inclusion/exclusion criteria and the characteristics of the patients recruited to participate in the study.

To be eligible, participants had to meet several criteria. These included being at least 18 years old; being capable of reading and signing an informed consent document; being psychologically and physically able to tolerate conventional restorative procedures; being free from high risk of tooth decay, periodontal disease, or pulp disease; having teeth with satisfactory previous restorations; requiring esthetic improvement for at least 4 front teeth in the upper jaw with multiple diastemas; not having any allergies to resin-based materials; not nursing or pregnant; and being willing to attend follow-up examinations as specified by the researchers. The study excluded patients with bruxism, temporomandibular disorders, and parafunctional behaviors. A total of 28 patients aged 18 to 30 (19 females, 9 males, mean age 26 years) were enrolled between August 2021 and January 2024. Participants received 120 laminate veneers. Fourteen of them received DC (n = 60), and another fourteen received IC (n = 60), as Figure 1 shows. Possibilities for alternative treatments were discussed. Every patient gave informed consent as required by the university’s ethical committee. The study was registered on Clinicaltrials.gov accessed on 10 September 2023 (identifier: NCT06377423).

### 2.4. Sample Size Calculation

In a prior study by Ozturk et al. [25], the 2-year follow-up resulted in an overall survival percentage of 91.2%. The null hypothesis that the failure rate for control and experimental subjects was equal with probability (power) 0.8 was rejected with a sufficient sample size of 108 IC laminate veneers (54 in each group). To account for a 10% dropout rate, a sample size of 120 subjects (60 in each group) was determined. For this test of the null hypothesis, the type I error probability was 0.05. The sample size was calculated by PS (power and sample size) using nQuery Advisor version 7.0.

### 2.5. Calibration Procedures for Clinical Evaluation

Two examiners who did not assist with restoration placement received training for the assessment procedure in July 2021. Before the evaluation process, an intra-examiner and inter-examiner Kappa agreement of at least 90% was necessary [23].

### 2.6. Clinical Procedures for IC Veneers

Both digital photographs and stone casts were used in the treatment planning process. In the dental laboratory, shade was evaluated using several shade tabs under standard settings (6500 K, 8 light intensity, Longlife, Aura, The Netherlands). The plaster model was used to create a wax setup using the mock-up method. The patient’s expectations were assessed, and discussions regarding modifying the teeth’s form and position were conducted using the wax setup.

**Figure 2:**
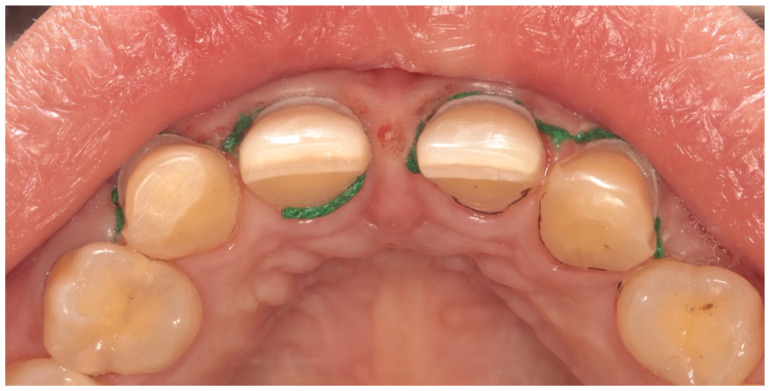
Occlusal view showing equi-gingival chamfer chamfer finish line (0.5–0.7 mm) at the cervical region.

For minimal preparation, a powerful illumination intensity from a connected light source (EyeMag Light II, Carl Ziess Meditec Ag, Jena, Germany) was used with a magnifying dental loupe with a working distance of 40 cm and magnification of 4.3× (EyeMag Pro F, Carl Ziess Meditec Ag, Germany). Preparation depths were marked at the incisal edge with a depth marker bur (707 C, Porcelain Veneer Prep-set, Intensive, Viganello-Lugano, Switzerland). The labial surfaces were marked with a depth cut bur (834-004, S4 STD, Intensive, Viganello-Lugano, Switzerland) and then reduced by 0.3–0.5 mm. Uniform preparations were carried out using tapered round-ended diamond burs (235A C), and then margins were finished and polished with a specially designed bur (FG 4307N). A chamfer finish line (0.5–0.7 mm) was made at the cervical region to ensure good periodontal health as Figure 2 shows.

To minimize stress concentration, all internal angles were rounded and smoothed. The incisal edge and palatal surface were positioned at a right angle to form a butt joint on the palatal surface. Next, impressions were created with a polyether impression substance (Impregum, 3M ESPE, St. Paul, MN, USA). Auto-polymerized bis-acryl Protemp 4 (3M ESPE, Seefeld, Germany) and the spot-etch technique were used to create temporary chair-side veneers.

The veneers were made using lithium-disilicate glass ceramic (IPS e.max Press, Ivoclar Vivadent) combined with a low-fusing nano-fluorapatite glass ceramic (IPS e.max Ceram, Ivoclar Vivadent). The veneers were characterized using a layering technique as recommended by the manufacturers.

Following the temporary restoration’s removal, the clinical assessment involved matching the shade of the veneers, contour, form, marginal adaption, and contacts. Try-in pastes (Variolink Try-in paste, Ivoclar Vivadent, NY, USA) were used to determine the cement’s color. Table 2 explains the bonding surface methods using light-cured resin cement (Variolink Veneer, Ivoclar Vivadent) for the porcelain and tooth surfaces. Following these processes, veneers were positioned, and extra luting cement was cleaned away using a brush, dental floss, and hand instruments. Additionally, all cementation procedures were performed under rubber dam isolation. Veneers were cervically precured for 5 s, before final curing to fully eliminate any extra cement in the interproximal and cervical regions. Then, every surface received a final curing session for 40 s. Using silicon polishers (Astropol FP, HP, Ivoclar Vivadent) and interproximal strips (Soft-Lex Finishing Strips, 3M ESPE, St. Paul, MN, USA), the veneer margins were polished between 7.500 and 10.000 rpm while they were immersed in water. Ultimately, protrusive and lateral mandibular movements were examined to confirm the occlusion.

### 2.7. Clinical Procedures for DC Veneers

For DC, the relative adhesive strategy was combined with the nanofilled composite resin (Estelite Asteria, Tokuyama Dental, Japan). Using button composite sampling and cross-polarization dental photography, an appropriate single shade was chosen. A thick rubber dam (Thick Blue, Nictone, Romania) was used to isolate the maxillary anterior teeth, and dental floss or specialized rubber dam retraction clamps (Black Line, Hu-Friedy, Chicago, IL, USA) were used to retract the teeth. In general, no preparations were made for diastema closure (non-prep); nonetheless, the minimally invasive dentistry concept was applied as a preparatory guideline in certain cases.

Before veneering, the teeth underwent a cleaning process using a rubber cup and a slurry of pumice and water then with a glycine powder blasting device (PROPHYflex KaVo Dental AG, Biberach, Germany) to remove the aprismatic enamel layer. Next, the teeth’s enamel surfaces were treated with 37% phosphoric acid gel for 30 s, followed by rinsing and drying. The Bond Force II self-etch adhesive system (Tokuyama Dental, Japan) was used to treat the etched surface. It was applied, rubbed for 15 s, dried with gentle air pressure for 15 s, and then light-cured for 10 s. The restorations were performed using the monochrome free-hand layering approach. Transparent strips, metal strips, or silicone indexes were employed in conjunction with the free-hand approach as needed. Nevertheless, a special restorative instrument kit (LM-Arte Set, LM Dental, Parainen, Finland) [26] and composite brushes (Tokuyama Brush no. 24, Tokuyama Dental, Japan) [26] were used.

**Figure 3:**
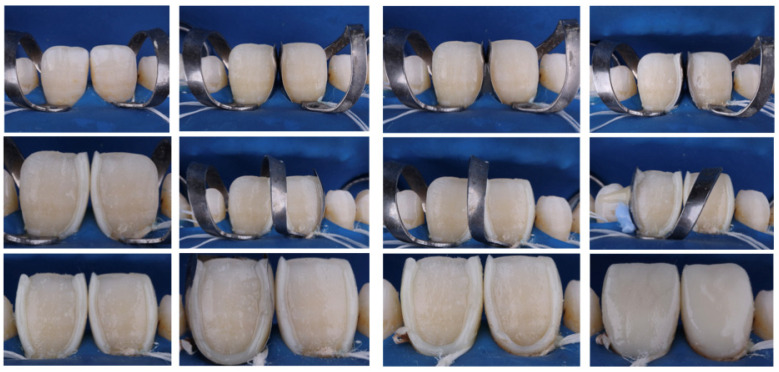
Showing photographs of a representative case following the clinical protocol for the build-up of composite restorations.

A monochromatic restoration was created using homogeneous spherical fillers and the chameleon effect of composite resin (a particular body shade of Estelite Asteria). To prevent the formation of an oxygen inhibition layer, the final labial surface layers of the restorations were polymerized under a glycerin gel cover (Air Barrier, GC Corp., Tokyo, Japan). The monochromatic building was completed progressively in <2 mm levels, and each layer was polymerized for 20 s. Photographs were taken along the buildup procedures to ensure the composite’s optimal thickness to avoid protrusion. The final occlusion was adjusted in the mandible’s protrusive and lateral movements. A step-by-step photograph of a representative case following the clinical protocol for the build-up of composite restorations is presented in Figure 3.

For polishing, 3 different levels of coarseness of interdental polishing strips (Epitex Strips, GC Corp.) were employed: medium #500, fine #800, and extra-fine #1200. The incisal, cervical, labial, and palatal embrasures were adjusted according to the manufacturer’s instructions. This was completed using aluminum-oxide-embedded polishing discs (Sof-Lex, 3M) with 3 different grain sizes (medium, 40 μm; fine, 24 μm; super-fine, 8 μm). The adjustments were made in dry conditions and at a 12,000-rpm speed.

A diamond Perio-bur #831 (Komet Dental, Lemgo, Germany) was used to create the restorations’ fundamental anatomy. The procedure was conducted at a speed of 12,000 revolutions per minute with water coolant’s assistance. To achieve microsurface morphology (secondary anatomy), a blue diamond bur (Diatech, Heerbrugg, Switzerland) was used horizontally at a speed of 5000 rpm without water cooling. The labial and palatal surfaces were polished using flexible diamond-impregnated spiral wheels (EVE Ernst Vetter GmbH; Pforzheim, Germany) in a counterclockwise orientation at 5000 rpm (without water cooling) according to the manufacturer’s instructions. During the 1-week visit, the veneers were subjected to surface repolishing to ensure that polymerization was fully accomplished.

### 2.8. Clinical Evaluation

Assessments were conducted using information gathered from the patients’ medical and dental histories in addition to macro photographs of the restorations. Crop-frame (D7200, Nikon, Tokyo, Japan), macro (105 mm VR, Nikon), twin flashlight (R1C1, Nikon), twin-flash mounted bracket (Owlbrcket C, Torun&Torun, Ankara, Turkey), and cross-polarized filters (PolarEyes, PhotoMed, CA, USA) were all part of the photography package. Debonding, fracture to failure, and caries were regarded as absolute failures. Inquiries on potential postoperative complaints were also made of the patients. After a week (baseline), patients were called back to reexamine the gingival margins, proximal contacts, and occlusion.

**Figure 4:**
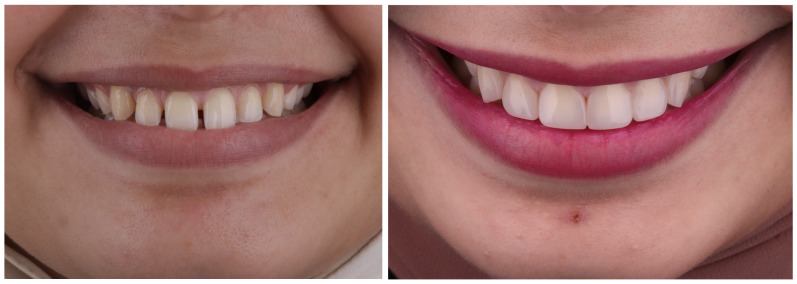
Pre- and post-operative (baseline) photos for direct composite veneers in multiple diastema closure case with good clinical outcome.

**Figure 5:**
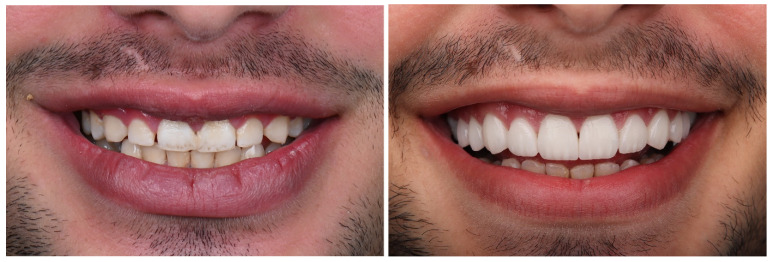
Pre- and post-operative (baseline) photos for indirect ceramic veneers in multiple diastema closure case with good clinical outcome.

Two experienced restorative dentists, oblivious to the study’s objective apart from the one who carried out the restoration treatments, reevaluated the veneers at baseline and after 6, 12, and 24 months based on modified United States Public Health Service (USPHS) criteria [23] as Table 3 shows. Using a magnification dental loupe along with powerful lighting, the veneers were examined visually. Figure 4 and Figure 5 show pre- and postoperative dental photographs of DC and IC laminate veneers.

### 2.9. Statistical Analysis and Data Interpretation

SPSS software, version 25 (SPSS Inc., PASW Statistics for Windows version 25, Chicago, IL, USA), was used to analyze the data. Quantitative data were described using the median (minimum and maximum) for non-normally distributed data, whereas qualitative data were described using percentages and numbers. The outcomes obtained were deemed significant at the (*p* ≤ 0.05) level. Fisher’s exact test and chi-squared test were used to compare qualitative data between groups. Assumptions include a random selection of data, categorical data, mutually exclusive categories, single data contribution, independence of study groups, and specific cell expected frequency. Moreover, the Cochrane test was used to compare various follow-up durations with assumptions that responses are binary and from k-matched samples while the subjects are independent of one another and were selected at random from a larger population. The McNemar and Stuart–Maxwell tests were used to compare every 2 readings assuming that the pair of outcomes are collected on independent participants. The Kaplan–Meier curve was used to assess time to event using fracture as the event with assumptions of proportional hazard.

## 3. Results

Twenty-eight patients had treatment involving the application of 120 veneers. The patients were then followed up for a period of 2 years. Every patient was given a subsequent assessment after 1 week (baseline) and 6, 12, and 24 months. There were no instances of patient loss over the 2-year follow-up period. All restorative treatments were executed precisely according to the plan, without any additional alterations. The agreement between the examiners was found to be satisfactory based on the overall Cohen’s κ statistics. Specifically, the agreement was 0.95 at the baseline, 0.94 at 12 months, and 0.95 at 24 months. Table 4 provides a summary of the total count of restorations and summaries of USPHS evaluations during the baseline and 24-month follow-up phases. After 24 months, 4 restorations (6.6%) in the DC group exhibited moderate chippings and cracks, necessitating repairs. Nevertheless, the IC group experienced a total of three instances of absolute failure, accounting for 5% of the group. These failures manifested as debonding in one case and fracture in two cases. Debonding refers to a total breakdown of the adhesive bond between the tooth and the luting cement. This occurred 23 months after the cementation process. No patients exhibited any signs of tooth fractures, wear of the antagonist or restorations, or caries.

**Figure 6:**
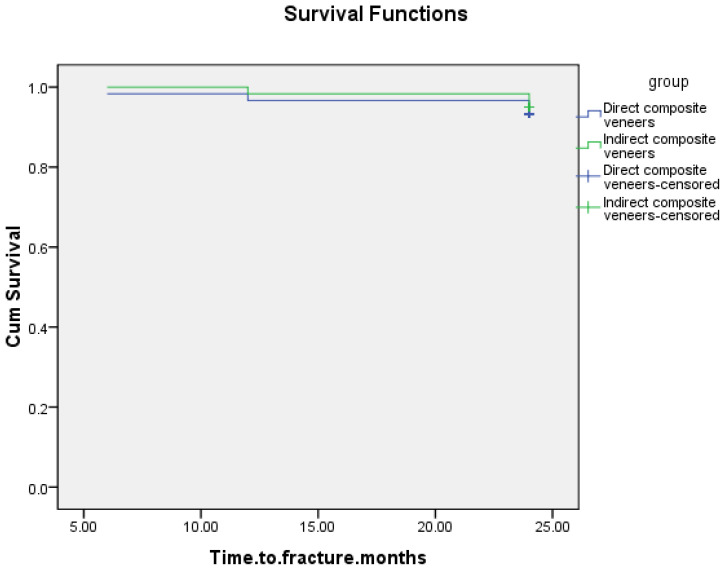
Survival curve of direct composite veneers and indirect ceramic veneers based on material (Estelite Asteria: 93.3%, n = 60; evens n = 4; IPS emax press: 95%, n = 60; events n = 4).

According to Figure 6, the DC (93.4%) and IC (95%) survival rates did not differ significantly (*p* > 0.05; Kaplan–Meier, Log Rank [Mantel-Cox], CI = 95%). The Kaplan–Meier analysis revealed an overall survival rate of 94.2%. Because of the lack of substantial differences between the groups, it was not possible to construct hazard ratios.

**Figure 7:**
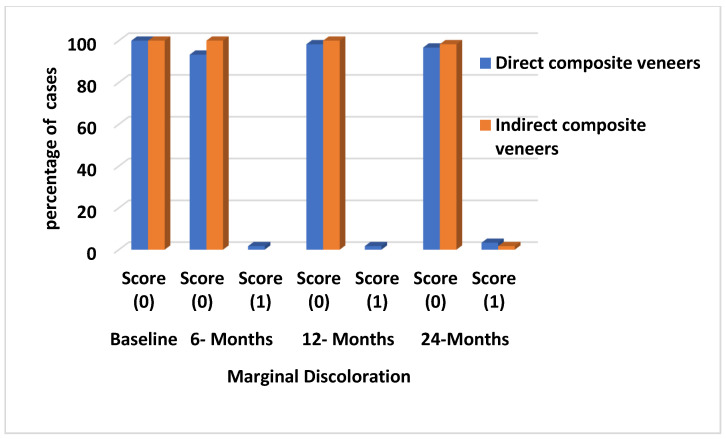
Charts representing marginal discoloration in both groups along the follow-up period (percentage of cases scores 0, 1 at baseline, 6 months, 12 months, and 24 months).

**Figure 8:**
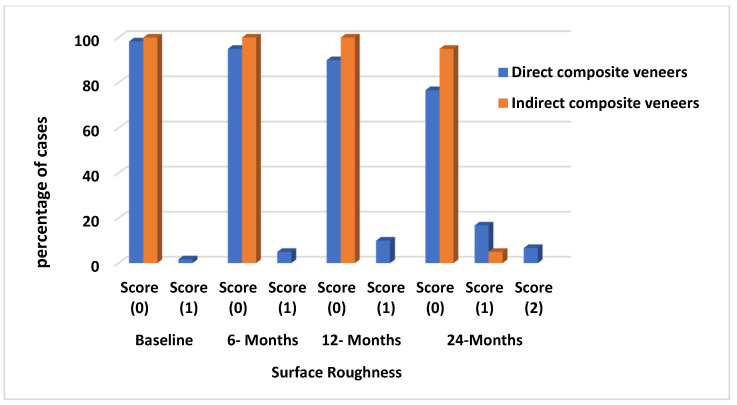
Charts representing surface roughness in both groups along the follow-up period (percentage of cases scores 0, 1 at baseline, 6 months, 12 months, and 24 months).

A minor discrepancy in color and shade was noted in both DC (n = 2, color match, score 2) and IC (n = 2, color match, score 2), but it was not statistically significant (*p* = 0.496). The marginal discoloration in DC was found to be significantly different from IC group (*p* = 0.04). Slight staining (n = 7, marginal discoloration, score 1) and evident staining at the margins (n = 4, marginal discoloration, score 2) were observed. Nevertheless, the IC exhibited minimal staining at its margins (n = 2, marginal discoloration, score 1) as Figure 7 shows. Concerning roughness, there was a significant difference (*p* = 0.01) between the 2 groups. The DC group had a higher frequency of slightly rough surfaces (n = 10, surface roughness, score 1) and surfaces with obvious roughness that could not be polished away (n = 4, surface roughness, score 2) compared to the other group, across the entire recall period. Nevertheless, the IC exhibited a minor degree of roughness on its surfaces (n = 2, surface roughness, score 1) as Figure 8 shows. Twenty-two teeth exhibited postoperative sensitivity at the baseline for both groups, and there was no significant difference between them (*p* = 0.6). After 2 weeks, all sensitivities after the treatment procedure were completely resolved.

## 4. Discussion

In this study, we assessed the clinical efficacy of DC and IC veneers bonded to the enamel tooth structure for a duration of 2 years. Longitudinal studies have various drawbacks, such as the discontinuation of specific dental materials and the loss of a portion of patients over time. All patients were successfully followed up, and the materials used are still accessible. Thus, the findings of this study were disclosed following a comparatively brief period. The findings indicated that DC and IC are favorable dental restorations, enduring 2 years of clinical use and exhibiting promising survival rates.

The survival rates of ceramic veneers seen in this investigation are similar to those reported in earlier midterm clinical studies, which indicate survival rates of 94% [1] and 93.5% [27] after 2.5 and 3 years, respectively. Hence, the study recorded a comparatively low number of failures, specifically three absolute failures with an overall survival rate of 94.2%. The low failure rates indicated that both the clinical protocol followed and the materials employed are dependable. Nevertheless, a comprehensive and extended monitoring of an increased number of restorations is necessary.

Our results corroborated the earlier 5-year follow-up findings of Feese et al. [28] and Peumans et al. [21]. After two concerning composite veneers, except for failed restorations, the majority of restorations demonstrated outstanding quality with improved functional and esthetic outcomes after 2 years of clinical care. There were no noticeable variations in DC and IC laminate veneers’ longevity when used in multiple diastema closure cases over 2 years. Therefore, the null hypothesis was deemed valid and accepted.

Within this investigation, a single restoration (1.6%) in the IC group exhibited debonding, while two fractures (3.3%) were seen. In the DC group, four restorations (6.6%) displayed moderate chippings and cracks after 24 months. Fractures are commonly cited as the primary reason for composite and ceramic veneers’ clinical failure [8,10,13]. Peumans et al. (2%) [10], Magne et al. (0%) [29], Korkut et al. (6.3%) [13], and Guess and Stappert (2.3%) reported the percentage of incidences of clinically unsatisfactory fractures [8]. Three instances of absolute failure were observed in this investigation, accounting for 5% of the cases. This finding aligns closely with the outcomes reported in other clinical follow-up studies.

According to Heintze et al. [22], restorations that involve the incisal edge have a higher likelihood of failure compared to class III restorations. Our study found that a greater number of surfaces were treated with composite material for closing diastemas and veneering tooth surfaces, leading to the creation of larger restorations. Therefore, the lower average survival rate (94.2%) for restorations may be attributed to the larger dimensions of the restorations, which is considered a risk factor for potential failure [20,22,30].

The DC veneers most commonly experienced relative failures in terms of adaptation, marginal discoloration, and surface roughness. Anticipated alterations of this nature were foreseen; however, the magnitude and rapidity of the transformation were uncertain. Resin composites demonstrated equivalent color matching to ceramic laminate veneers. Both the DC and IC showed a little discrepancy in color and shade, with a color match score of 2 (n = 2). The findings of our study align with those of Paravina et al. [31], who observed that composite resins containing nano-spherical fillers exhibited the most accurate color matching.

The surface roughness became apparent when the laminate surfaces dried out naturally to eliminate the thin layer of saliva, as per the evaluation specifications based on USPHS criteria. Although the surface roughness increased, it did not necessarily result in a color change. Perhaps it was because of this factor that none of the patients expressed dissatisfaction with the alterations to the surface, and a few were even oblivious to them.

Within the dentistry field, the deterioration of the surface is seen as a justification for replacing resin composite restorations. Deterioration of resin-based materials over time can result in the leaching of their components as well as the swelling of the cross-linked resin structure [32,33]. Given that DC veneers are not applied to the occlusal surfaces, the application and polishing operations’ impact becomes more significant [34]. It was anticipated that evaluating the nano-filled composite would improve surface qualities because of its high filler content and photopolymerization methods [34]. A recent study found that resin-based products have a principal resin matrix on the exterior surface following polymerization, rather than fillers [34].

One of the factors contributing to the aging of direct resin composite surfaces is the biofilm effect, which occurs when the microbial compositions on the surfaces are similar [34]. This effect may be due to variations in the concentrations of positively charged inorganic components on the composite surfaces [35]. Although previous research, both in vitro [35,36,37] and clinical studies [9,38], has shown that ceramic materials may experience considerable surface deterioration, the lithium-disilicate-based ceramic employed in this investigation maintained smooth surfaces throughout the observation period (n = 2, surface roughness, score 1).

In the DC, there were a few minor voids and defects identified at the margins: 3 out of 60 with DC and 1 out of 60 with IC according to the USPHS criteria; adaption, score 1. Additionally, there was both faint and evident staining (n = 11, marginal discoloration, scores 1, 2) in the DC. Despite this, IC displayed minor staining at the margins (n = 2, marginal discoloration, score 1) up until the point where the final recall was performed. In other research, these problems were found at not only the interface between the laminate and the prior restorations but also the interfaces between the tooth and the laminate [10]. This was the specific location where the flaws were discovered. The margins in these areas were hidden and could not be evaluated because the preparation margins were extended to the proximal sites. This prevented the margins from being evaluated. As a result, flaws, minor voids, and marginal stains were found mostly along the incisal or cervical margins throughout the majority of the cases. Previous research has indicated that the occurrence of adaption defects has been found to rise from 1.2% after 6 years to 7.9% after 12 years [6,10].

Postoperative sensitivity (10 out of 60 with DC and 12 out of 60 with IC, USPHS criteria; sensitivity scores 1, 2) was observed at baseline. Therefore, there was no noticeable significant difference observed between the DC and IC groups. Nevertheless, there were significant differences observed in postoperative sensitivity over time in both groups.

The results of this study align with the findings of Marco et al. [27], who reported that there is no noticeable difference in postoperative sensitivity between indirect resin composite and IC restorations. Furthermore, he demonstrated that the use of indirect resin composite and IC veneers for tooth restoration resulted in a substantial decrease in postoperative sensitivity, as compared to the initial sensitivity level, even after a period of 3 years [27]. Furthermore, this study examined the occurrence of postoperative sensitivity for 2 years and confirmed a substantial decrease in postoperative sensitivity throughout the review period, consistent with prior research findings [13,27].

A significant drawback of this clinical study is that the observation duration of 2 years may be insufficient to detect any changes. Hence, conducting a comprehensive and extended clinical assessment would be more effective in evaluating the disparity between DC and IC laminate veneers. Therefore, the veneers from the current study are being monitored for an extended period, considering both absolute and relative failure.

## 5. Conclusions

Taking into consideration this clinical investigation’s preliminary findings, the following can be concluded: when comparing absolute failures, there was no statistically significant difference in the survival rates of the DC and IC veneers, which were examined for up to 2 years. The composite veneer material exhibited a higher frequency of surface quality alterations, perhaps necessitating increased maintenance over time.

## 6. Clinical Relevance

Direct composite veneers can fulfill both esthetic and functional requirements, similar to indirect ceramic veneers. Nevertheless, in cases where it is recommended, anterior indirect ceramic laminate veneers may be favored over direct composite laminate veneers.

## Figures and Tables

**Table 1:** Restorative materials used in the present study.

Material	Description	Composition	LOT No.	Manufacturer
IPS e.max Press	Lithium disilicate	SiO_2_, Li_2_O, K_2_O, MgO, ZnO, Al_2_O_3_, P_2_O_5_, and other oxides.	M122766	Ivoclar Vivadent, Amherst, NY, USA
Estelite Asteria (Body shades)	Nano-filled spherical composite resin	Matrix: Bis-GMA, Bis-MPEPP, TEGDMA, UDMA Filler: Uniform supra- nano spherical silica and zirconia fillers (200 nm). 82 wt %, 71 vol %	W122	Tokuyama Dental, Tokyo, Japan
Variolink Veneer	Photopolymerized luting cement	Urethane dimethacrylate, decamethylene dimethacrylate, inorganic fillers, ytterbium trifluoride, initiators, stabilizers, pigments	P38751	Ivoclar Vivadent, Amherst, NY, USA
Monobond S	Silane coupling agent	1% 3-methacryloxypropyltrimethoxysailane, ethanol-/water-based solution	K30454	Ivoclar Vivadent, Amherst, NY, USA
Ceramic etching gel	Hydrofluoric acid	<5% hydrofluoric acid	1000001664	Ivoclar Vivadent, Amherst, NY, USA
Syntac Primer	Primer	Triethylene glycol dimethacrylate, polyethylene glycol dimethacrylate, maleic acid, acetone	K12455	Ivoclar Vivadent, Amherst, NY
Syntac adhesive	Adhesive	polyethylene glycol dimethacrylate, glutaraldehyde	K30566	Ivoclar Vivadent, Amherst, NY
Heliobond	Bonding agent	Bis-GMA, triethylene glycol dimethacrylate, catalysts, stabilizers	K30325	
Total etch	Phosphoric acid	37% phosphoric acid	K17720	Ivoclar Vivadent, Amherst, NY
Try-in Paste	Glycerin	Glycerin, mineral fillers and dyes	7405123	Ivoclar Vivadent, Amherst, NY
Bond Force II	Self-etch adhesive agent (single bottle)	Hydroxyethyl methacrylate, bisphenol A di (2-hydroxy propoxy) dimethacrylate, camphorquinone, dibutyl hydroxy toluene, diphenyl (2,4,6-trimethylbenzoyl) phosphine oxide, mequinol, phosphoric acid monomer, propan-2-ol, water, triethylene glycol dimethacrylate	202	Tokuyama Dental, Tokyo, Japan

Abbreviations: Al_2_O_3_, aluminum oxide; Li_2_O, lithium oxide; K_2_O, potassium oxide; MgO, magnesium oxide; P_2_O_5_, phosphorus pentoxide; ZnO, zinc oxide, Bis-GMA, Bisphenol A diglycidil ether dimethacrylate; UDMA, Diurethane dimethacrylate; TEGDMA, Triethylene glycol dimethacrylate; phA-m, Phosphoric acid ester monomer.

**Table 2:** Surface conditioning protocol for the tooth and porcelain surface of indirect ceramic veneers.

Surface Treatment	Tooth Surface	Porcelain Inner Surface
Etching	Enamel: 37% phosphoric acid for 30 s Dentin: 37% phosphoric acid for 15 s	5% Hydrofluoric acid for 20 s
Adhesive	1- Apply Syntac Primer for 15 s 2- Apply Syntac adhesive for 10 s 3- Apply Heliobond for 10 s	1- Apply Monobond Silane coupling agent for 60 s 2- Apply Heliobond for 10 s

**Table 3:** List of modified United States Public Health Service (USPHS) criteria used for the clinical evaluations of the veneers.

Category	Score	Criteria
Adaptation	0	Smooth Margin
	1	All margins closed or possess minor voids or defects (enamel exposed)
	2	Obvious crevice at margin, dentin or base exposed
	3	Debonded from one end
	4	Debonded from both ends
Color match	0	Very good color match
	1	Good color match
	2	Slight mismatch in color or shade
	3	Obvious mismatch, outside the normal range
	4	Gross mismatch
Marginal Discoloration	0	No discoloration evident
	1	Slight staining, can be polished away
	2	Obvious staining, cannot be polished away
	3	Gross staining
Surface roughness	0	Smooth surface
	1	Slightly rough or pitted
	2	Rough, cannot be refinished
	3	Surface deeply pitted, irregular grooves
Fracture of restoration	0	No fracture
	1	Minor crack lines over restoration
	2	Minor chippings of restoration (1/4 of restoration)
	3	Moderate chippings of restoration (1/2 of restoration)
	4	Severe chippings (3/4 restoration)
	5	Debonding of restoration
Fracture of tooth	0	No fracture of tooth
	1	Minor crack lines in tooth
	2	Minor chippings of tooth (1/4 of crown)
	3	Moderate chippings of tooth (1/2 of crown)
	4	Crown fracture near cementum enamel line
	5	Crown-root fracture (extraction)
Wear of restoration	0	No wear
	1	Wear
Wear of antagonist	0	No wear
	1	Wear of antagonist
Caries	0	No evidence of caries continuous along the margin of the restoration
	1	Caries evident continuous with the margin of the restoration
Postoperative sensitivity	0	No symptoms
	1	Slight sensitivity
	2	Moderate sensitivity
	3	Severe pain

**Table 4:** Summaries of USPHS evaluations at baseline and final follow-up.

Criteria	Score	Baseline			Final Evaluation		
		Direct Composite Veneers (n = 60)	Indirect Ceramic Veneers (n = 60)		Direct Composite Veneers (n=60)	Indirect Ceramic Veneers (n = 60)	
Adaptation of Restoration	0	59	60	*p* = 1	57	59	*p* = 0.505
	1	1	–		3	1	
	2	–	–		–	–	
	3	–	–		–	–	
	4	–	–		–	–	
Color Match	0	60	60	*p* = 1	58	58	*p* = 0.496
	1	–	–		–	–	
	2	–	–		2	2	
	3	–	–		–	–	
	4	–	–		–	–	
Marginal Discoloration	0	60	60	*p* = 1	49	58	*p* = 0.04 *
	1	–	–		7	2	
	2	–	–		4	–	
	3	–	–		–	–	
Surface Roughness	0	59	60	*p* = 1	46	57	*p* = 0.01 *
	1	1	–		10	3	
	2	–	–		4	–	
	3	–	–		–	–	
Fracture of Restoration	0	60	60	*p* = 1	56	57	*p* = 1
	1	–	–		–	–	
	2	–	–		–	2	
	3	–	–		3	–	
	4	–	–		–	–	
	5	–	–		–	1	
Fracture of Tooth	0	60	60	*p* = 1	60	60	*p* = 1
	1	–	–		–	–	
	2	–	–		–	–	
	3	–	–		–	–	
	4	–	–		–	–	
	5	–	–		–	–	
Wear of Restoration	0	60	60	*p* = 1	60	60	*p* = 1
	1	–	–		–	–	
Wear of Antagonist	0	60	60	*p* = 1	60	60	*p* = 1
	1	–	–		–	–	
Caries	0	60	60	*p* = 1	60	60	*p* = 1
	1	–	–		–	–	
Post-operative Sensitivity	0	50	48	*p* = 0.676	60	60	*p* = 1
	1	4	3		–	–	
	2	6	9		–	–	
	3	–	–			–	

Abbreviations: *, significant difference.

## Data Availability

The original contributions presented in the study are included in the article, and further inquiries can be directed to the corresponding author.
